# Supplementary material for: COVID-19 Induces Greater NLRP3 Inflammasome Activation in Obese Patients than Other Chronic Illnesses: A Case–Control Study
Source: Int J Mol Sci. 2025 Feb 12;26(4):1541. doi: 10.3390/ijms26041541 (PMC11855377; doi:10.3390/ijms26041541)
Supplement: Supplementary file 1 [file ijms-26-01541-s001.zip › Supplementary Table S1.pdf]

Supplementary Table S1: Clinical and Epidemiological Data of Group A

PARTICIPANT	AGE	SEX	COMORBIDITIES	MECHANICAL VENTILATION TIME
1	80 years	Male	Previous CAD and CABG, HFrEF, hypertension, dyslipidemia, chronic AF, OSAHS, COPD, chronic venous insufficiency, epilepsy.	20 days
2	57 years	Male	SAH, DM, anxiety disorder	10 days
3	75 years	Female	Asthma, COPD, Former smoker, SAH, AMI with CHF, DM, hypothyroidism, MP implant in February 2020 due to AMI with CAVB, CKD, Dyslipidemia.	9 days
4	64 years	Male	Non-dialysis CKD, history of 2 kidney transplants (25 years and 9 years ago), SAH, CAD with previous PCI.	4 days
5	57 years	Male	SAH	37 days
6	57 years	Male	SAH, DM, Generalized anxiety disorder.	24 days
7	86 years	Male	SAH, pre-DM, BPH, Dyslipidemia.	3 days
8	72 years	Female	SAH, DM, Hypothyroidism	27 days
9	72 years	Female	SAH, Dyslipidemia.	19 days
10	54 years	Male	SAH, Cardioembolic Ischemic Stroke (PFO), Hypothyroidism	8 days
11	57 years	Male	Denies	12 days
12	79 years	Male	DM, SAH	16 days
13	51 years	Male	SAH, Chest lymphoma treated in 2019 in remission, Former smoker	15 days
14	77 years	Male	Parkinson's disease, stroke (lacunar infarction on the right globus pallidus January/21)	18 days

Legend: **CAD:** Coronary Artery Disease; **CABG**: Coronary Artery Bypass Grafting; **HFrEF**: Heart Failure with Reduced Ejection Fraction; **AF**: Atrial Fibrillation; **OSAHS**: Obstructive Sleep Apnea Hypopnea Syndrome; **COPD**: Chronic Obstructive Pulmonary Disease; **SAH**: Systemic Arterial Hypertension (Hypertension); **DM**: Diabetes Mellitus; **AMI**: Acute Myocardial Infarction; **CHF**: Congestive Heart Failure; **MP**: Pacemaker; **CAVB**: Complete Atrioventricular Block; **CKD**: Chronic Kidney Disease; **BPH**: Benign Prostatic Hyperplasia; **PFO**: Patent Foramen Ovale.
