# Supplementary material for: COVID-19 Induces Greater NLRP3 Inflammasome Activation in Obese Patients than Other Chronic Illnesses: A Case–Control Study
Source: Int J Mol Sci. 2025 Feb 12;26(4):1541. doi: 10.3390/ijms26041541 (PMC11855377; doi:10.3390/ijms26041541)
Supplement: Supplementary file 1 [file ijms-26-01541-s001.zip › Supplementary Table S2.pdf]

Supplementary Table S2: Clinical and Epidemiological Data of Group B

PARTICIPANT	AGE	SEX	COMORBIDITIES	CAUSE OF DEATH	MECHANICAL VENTILATION TIME
1	65 years	Male	Hypertension	Ruptured dissecting aortic aneurysm in the abdominal segment, hemoperitoneum, hemomediastinum.	1 day
2	75 years	female	Hypertension; Heart Failure with EF 39%	Deep Vein Thrombosis + Pulmonary Embolism	7 days
3	79 years	Female	Endometrial cancer under treatment for 3 years	Endometrial Adenocarcinoma + Pelvic Peritoneal Carcinomatosis + Pulmonary Metastases + Deep Vein Thrombosis in the LLE + Pulmonary Embolism	2 days
4	58 years	Male	Alcoholic cirrhosis - Child B; Hepatic Transplantation	Hemorrhage + Orthotropic Liver Graft with Signs of Necrosis	1 day
5	66 years	Male	Coronary Artery Disease; COPD	Severe Systemic Atherosclerosis + Severe Acute Pulmonary Edema	1 day
6	42 years	Male	Hypertension; Dyslipidemia; Former Smoker	Acute Infarction in the Right Atrium + Severe Pulmonary Edema	1 day
7	79 years	Female	Hypothyroidism; Hypertension; Depression; Stroke 4 years ago without sequelae; Nephrectomy 9	Infectious Endocarditis of the Aortic Valve + Severe Pulmonary Edema with thrombi in the	9 days

			years ago due to renal cancer.	organization in the LLE	
8	47 years	Male	Chronic Myeloid Leukemia	Chronic Myeloid Leukemia + Severe Graft-versus-Host Disease (Grade III)	1 day
9	52 years	Male	Alcoholic cirrhosis - Child B	Hemoperitoneum	

Legend: **EF**: Ejection Fraction; **COPD**: Chronic Obstructive Pulmonary Disease; **LLE**: Left Lower Extremity.
