# Supplementary material for: COVID-19 Induces Greater NLRP3 Inflammasome Activation in Obese Patients than Other Chronic Illnesses: A Case–Control Study
Source: Int J Mol Sci. 2025 Feb 12;26(4):1541. doi: 10.3390/ijms26041541 (PMC11855377; doi:10.3390/ijms26041541)
Supplement: Supplementary file 1 [file ijms-26-01541-s001.zip › Supplementary Table S3.pdf]

Supplementary Table S3: Antibodies used for the immunohistochemical assay

Antibody	Type	Clone/Code	Dilution	Manufacturer
Anti-IL-6	Monoclonal/Mouse	Ab9325	1:400	Abcam
Anti-IL-8	Monoclonal/Mouse	807	1:200	Abcam
Anti-TNF-α	Monoclonal/Mouse	TA20	1:50	Santa Cruz
Anti-NALP	Policlonal/Goat	ab4207	1:100	Abcam
Anti-IL-18	Policlonal/Rabbit	EC9312	1:200	Elabscience
Anti-IL-1β	Policlonal/Rabbit	A16288	1:800	ABclonal
Anti-Gasdermin D	Policlonal/Rabbit	A18281	1:100	ABclonal
Anti-ASC	Policlonal/Rabbit	sc-33796	1:200	Santa Cruz
Anti-NF-Kb	Policlonal/Rabbit	Ab7972	1:400	Abcam
Anti-CASP 1	Policlonal/Rabbit	ab189796	1:200	Abcam
Anti-CASP 9	Policlonal/Rabbit	PAA627Hu01	1:100	Cloud-Clone
